# Development of Non Expensive Technologies for Precise Maneuvering of Completely Autonomous Unmanned Aerial Vehicles †

**DOI:** 10.3390/s21020391

**Published:** 2021-01-08

**Authors:** Luca Bigazzi, Stefano Gherardini, Giacomo Innocenti, Michele Basso

**Affiliations:** 1 Dipartimento di Ingegneria dell’Informazione (DINFO), Università di Firenze, via di Santa Marta 3, 50139 Firenze, Italy; luca.bigazzi@unifi.it (L.B.); giacomo.innocenti@unifi.it (G.I.); michele.basso@unifi.it (M.B.); 2 Dipartimento di Fisica e Astronomia & LENS, Università di Firenze, via G. Sansone 1, 50019 Sesto Fiorentino, Italy

**Keywords:** aircraft navigation, automatic control, computer vision, sensor fusion, unmanned aerial vehicles

## Abstract

In this paper, solutions for precise maneuvering of an autonomous small (e.g., 350-class) Unmanned Aerial Vehicles (UAVs) are designed and implemented from smart modifications of non expensive mass market technologies. The considered class of vehicles suffers from light load, and, therefore, only a limited amount of sensors and computing devices can be installed on-board. Then, to make the prototype capable of moving autonomously along a fixed trajectory, a “cyber-pilot”, able on demand to replace the human operator, has been implemented on an embedded control board. This cyber-pilot overrides the commands thanks to a custom hardware signal mixer. The drone is able to localize itself in the environment without ground assistance by using a camera possibly mounted on a 3 Degrees Of Freedom (DOF) gimbal suspension. A computer vision system elaborates the video stream pointing out land markers with known absolute position and orientation. This information is fused with accelerations from a 6-DOF Inertial Measurement Unit (IMU) to generate a “virtual sensor” which provides refined estimates of the pose, the absolute position, the speed and the angular velocities of the drone. Due to the importance of this sensor, several fusion strategies have been investigated. The resulting data are, finally, fed to a control algorithm featuring a number of uncoupled digital PID controllers which work to bring to zero the displacement from the desired trajectory.

## 1. Introduction

In the last decade, research in multi-rotor Unmanned Aerial Vehicles (UAVs) drawn increasing interest and funding from both academic and industrial communities, thanks to their versatility, low-cost realization, and promising unexplored applications [1,2]. Indeed, UAV research led to technological advances in mechatronic servo-systems, microelectronics, and sensors, which, combined with novel economically affordable and high-performing micro-controllers and embedded boards, rapidly increased their general performance [3,4,5].

Notable examples of successful application of UAV technologies can be found in precision agriculture [6,7,8], where drones equipped with Real-Time Kinematic Global Navigation Satellite Systems (GNSSs-RTK) [9] are used to minimize human intervention. Other important applications, instead, are related to monitoring and patrolling. For example, drones are well suited to explore or inspect large environments and buildings [10,11,12,13] aiming, for instance, to 3D reconstruction. Moreover, drones with high maneuverability in small spaces fits both urban missions [14,15,16,17,18], such as monitoring road traffic, and rescue missions [19,20,21,22,23,24,25], such as patrolling dangerous zone after natural disasters. In many of these applications Vision-Based Navigation (VBN) algorithms are often used to improve accuracy in the positioning [26,27,28,29,30,31,32,33,34]. Film makers and video studios are successfully using UAVs for aerial shooting [35,36]. These drones are usually big, because they need to load heavy cameras and their gimbals. The size, the weight, and the large number of engines make them very stable, and so, under the driving of skillful pilots, they are able to accomplish high precision maneuvers of the order of tens of centimeters. On-board systems are only used for improving stability and assisted maneuvering, while the navigation performance is completely left to the pilot’s ability and sensitivity.

In the field of autonomous UAVs, the actual precision for outdoor applications is limited by the mostly used sensing technology, that is, by standard GNSS devices, which today are able to locate the drone within a one meter radius at the very best [37,38]. The development of novel applications is severely limited by this performance level, and, therefore, the demand for better technologies is growing by the day. When higher performance is required, then, a finer positioning system turns out to be necessary, as it already happens for acrobatic drones [39,40,41]. To the authors’ knowledge, in this case the most effective approach consists in exploiting a ground-assisted positioning system. For example, in indoor applications, the typical solution involves a large number of fixed cameras, which are able to detect special markers on the drone. A ground station processes the data from the cameras by a computer vision algorithm that generates a high-precision position estimate at high-frequency. Possibly, the cameras can be replaced by other ground localization systems without changing the overall paradigm [42,43,44,45,46,47,48,49,50]. The ground station either can send the computed information to the drone, or, more usually, can pilot directly the UAV exploiting this data. Whichever the case, this solution prevents a completely autonomous drone.

The degree of autonomy of a UAV is a non-secondary factor when developing new applications, because any necessary environmental set up represents a cost and a delay. Recent projects, indeed, are investigating solutions, which may lessen the limits of the actual technologies recurring to an extensive use of sensor fusion techniques [51,52,53]. Nevertheless, precision and complete autonomy are yet difficult features to get together.

**Figure 1:**
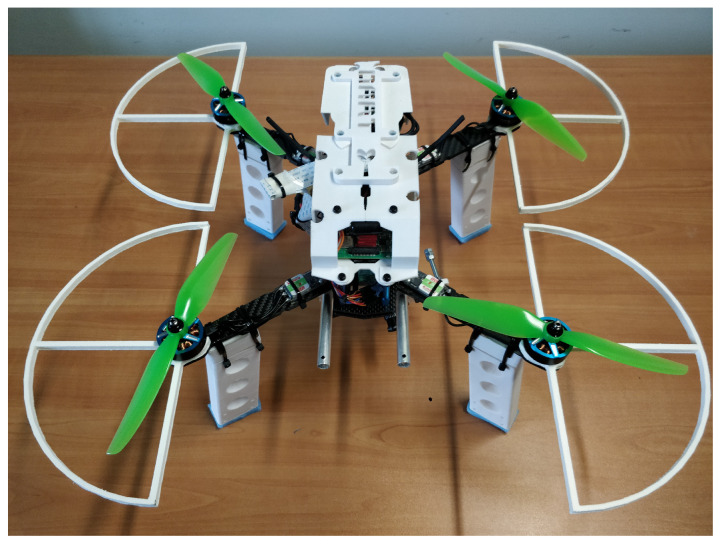
A picture of the Dart prototype.

In this work, following up on the conference paper in Reference [54], we present relevant advancements on our project “Dart”, an UAV—pictured in Figure 1—able to switch to a completely autonomous mode, sustained only by on-board systems, that is, without any ground assistance. The project scope is to obtain positioning and path following with centimetric precision by using only mass market technologies, in order to ascertain the gap between a completely autonomous ultra-high precision drones and actual commercial products with affordable prices. To this aim, Dart core has been designed to feature a high precision on-board vision-based positioning system made by assembling only mass market products, namely a small camera carried by a gimbal, an embedded electronic board, and an open-source computer vision library. The scenery captured by the camera is processed by a custom software, based on the computer vision library, which is designed to measure the drone position with respect to known markers. This information is then fused with data coming from a IMU to estimate the state of the drone. This system, therefore, acts as a virtual sensor that provides the automatic pilot with the information needed by the controllers designed to make the UAV follow the reference trajectory.

The paper is structured as follows. In Section 1.1 an overview of the Dart drone architecture is presented. In Section 2 each component of the hardware is illustrated, while Section 3 is devoted to describe the functionalities performed by the software modules, and the algorithms at their core. Section 4 describes the internal communications and the information flow, and also explains the schedule of each function. Finally, in Section 5 the results from the experimental tests are presented and discussed. Some final remarks and outlooks conclude the paper.

### 1.1. Overview of the Project & Article Novelties

Dart project is aimed to investigate if suitable modifications of standard technologies can be used to implement a completely autonomous high-precision drone, that is, a drone able to follow a reference trajectory with centimetric precision exploiting only on-board systems without any assistance from the ground, neither for sensing nor for computing. So far, this objective could be realized only on professional class drones with additional equipment as payload specifically conceived for the application in hand using proprietary software development kits. More recently, professional drones have improved their on-board resources, thus reducing the need of additional devices. Nevertheless, this approach comes with completely proprietary tools and more constraints on the development of new solutions. On a different perspective, it is also of great interest to measure how this kind of drones is far from mass market. Indeed, low-cost technologies are considered a key factor to boost the market of many innovative drone applications [55,56]. Considered this situation, we aim to develop an autonomous navigation device that can be introduced in any drone with sufficient payload. The adaptability of this solution will be guaranteed by a receiver by-pass, while on the cost side we will use only non expensive components to make its implementation sufficiently cheap. To test this novel technology, we will develop a custom drone, which is only meant to provide us with a flexible test bench.

The general architecture of the drone here presented consists in a 350-class UAV, where sufficient space and payload for some additional on-board devices can be obtained. Besides the basic electronics for engines and batteries, the drone features a 2.4 GHz remote receiver and a flight controller that has the sole task of stabilizing the attitude. Autonomy, meant as the capability to move along a desired trajectory without a driving pilot, comes from a digital signal mixer that allows the on-board navigation system to override the human commands and to replace the pilot, as long as this latter decides to get the control back. This way, the navigation system just substitutes the human pilot, and thus, they share the same interface with the drone. Due to its peculiar nature the automatic pilot is here referred to as “cyber-pilot”.

The concept behind Dart project is to separate the simpler tasks all concerning attitude stabilization from the so-called “smart” tasks related to complex navigation routines, such as the tracking of a complex trajectory in the 3D space. In our prototype, attitude stabilization is performed by a commercial board that constitutes the “low-level hardware”; instead, we consider the “high-level hardware” all the additional customized electronics that are necessary for autonomous navigation. This choice provides a two-fold advantage: (i) it requires the least number of modifications and the smallest customization; (ii) it allows us to focus only on the development of intelligent “high-level” algorithms for computer vision and tracking. Here, it is worth noting that in our drone, high- and low-level tasks are performed by two different hardware, since attitude stabilization—unlike high-level tasks—needs a control high-band to be successfully carried out. The first hardware is able to execute complex calculations at a lower rate (generally, a few hundred Hz) with respect to a standard micro-controller, while the second is optimized just to perform simple calculations but at a very high-frequency (in the order of kHz). Indeed, the attitude variation is much faster than the dynamics of its position. As it will be detailed in the following sections, experiments show the effectiveness of this solution. Finally, it is worth remarking that this functional stack, divided into low-level and high-level tasks, can be implemented by a modular hardware architecture, thus making the presented solutions very versatile and easy to implement in any other drone that can handle the payload required for the additional hardware.

In our drone prototype, the on-board navigation system has been developed on a popular programmable control board, which generates the maneuvering commands by computing only the information coming from an IMU and a camera mounted on a 3-DOF gimbal. The video stream of the camera is elaborated with a computer vision tool with the aim to infer the actual position of the drone with respect to the visible landmarks. Hence, the camera plays the role of a virtual position sensor. Data from this latter are then fused with those from the IMU to enhance the estimation of the drone state, that is, its position, speed and attitude. Eventually, the state estimate is compared with the reference path, and the displacement error used by the navigation system is thus generated to pilot the drone.

Dart drone has been designed, developed, and implemented employing very popular hardware components and electronic boards readily available on the market. This approach, however, does not mean the methods here presented to achieve autonomy are limited to this custom configuration or can not be applied to other off-the-shelf drone solutions. Rather, this choice has been functional for the preliminary investigation phase, when drone technologies and their interactions have been thoroughly analysed to point out where the additional devices for autonomy would provide the best trade off between effectiveness and cost. Moreover, commercial drones, even when conceived as developing platforms [57], cast serious restrictions to the designer’s creativity. In our case, for instance, the cyber-pilot idea is feasible in most drones, but it requires to implement a hardware by-pass on the receiver, which is not the most natural and obvious approach even in developer’s drones. Moreover, having complete access to the Dart hardware has been crucial during the first experiments, when the aim was in detecting any undesired effect between the additional devices of the cyber-pilot and the rest of the components.

**Figure 2:**
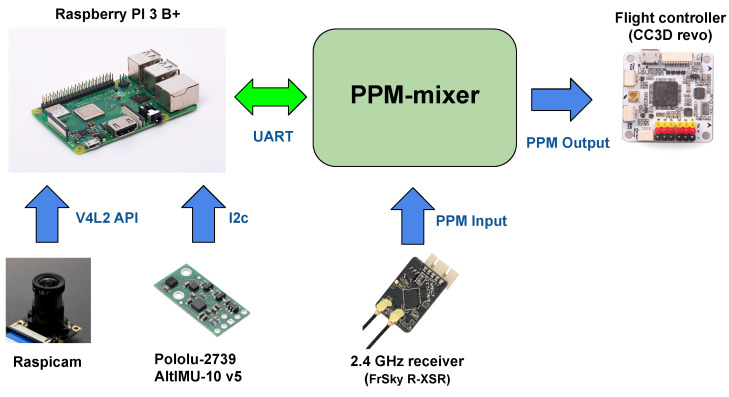
Hardware configuration and dependencies. The PPM-mixer board is an interface between the low-level flight controller and a Raspberry PI board, on which the high-level navigation software is implemented.

The graphical scheme of the above architecture is illustrated in Figure 2. Moving backward from the process output up to the inputs, the signal chain can be summarized as follows.

- Engines
- Flight Controller
- Signal Mixer
  – Receiver
    * Human pilot
  – Navigation System (Cyber-pilot)
    * Sensors
      · IMU
      · Stabilized Camera

In the next section, the hardware configuration of the drone will be detailed, and the core devices for developing the cyber-pilot technology (i.e., signal mixer and navigation system) will be (roughly) priced for the sake of a cost quantification.

## 2. Hardware Architecture

In this section the hardware configuration of our drone prototype Dart is introduced. In particular, the sensors units, with a special focus on the adopted computer vision system and the gimbal suspension, are discussed highlighting the main features that turn out critical for the precise positioning of the drone.

### 2.1. Mechanical Structure

The Dart body frame is mostly composed by laminated standard carbon fiber parts which improve the rigidity of the frame and reduce its weight. The body frame hosts other custom parts which have been 3D printed at low density, leaving empty spaces inside the material to make it lighter. These parts serve assembly of the additional hardware necessary for autonomous navigation and computer vision tasks.

### 2.2. Hardware Configuration

The architecture of the hardware is shown in Figure 2, where one can observe the interconnections between all components. A Raspberry PI 3 B+ board (Raspberry Pi Foundation, Cambridge, UK, ~$35) is connected with a Raspicam camera (price range $20–$40 depending on lens type) and a 6-DOF IMU, sensor units from which vision and inertial data are obtained. The navigation software running on the Raspberry board generates set-points for attitude and motors thrust, which are sent to the low-level flight controller realized on a CC3D Revo board (Open hardware). Currently, the low-level board has the sole task of stabilizing the attitude by following the set-points provided by the cyber-pilot (i.e., the high-level board) or by the human one. Between the Raspberry PI and the low-level, we employ a custom signal mixer board (Arduino Nano based on ATMEL AT328p micro-controller, Atmel Corporation, San Jose, CA, USA, ~$5) that we refer to as mid-level board, an important hardware novelty introduced in this paper.

**Figure 3:**
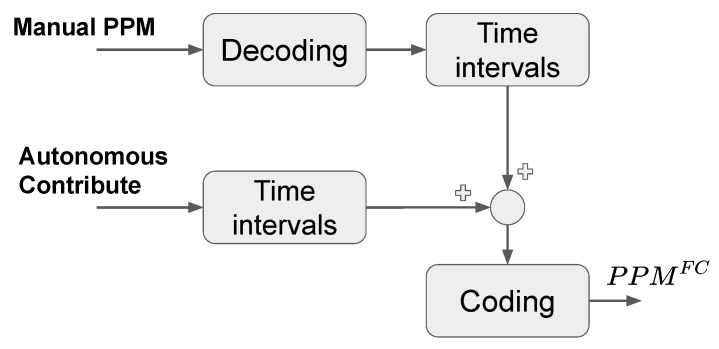
Functional scheme of the mixer board. The two input command signals, converted in time intervals, are merged into a single signal at the output of the board. We have named this operating logic as *Hybrid Mode*.

The mixer board takes in input the attitude set-points coming from the Raspberry (by using bidirectional UART protocol to convey the data stream) and the manual set-points coming from the 2.4 GHz receiver through the Pulse Position Modulation (PPM) protocol. As output the mixer generates a second PPM signal that is obtained by properly elaborating the inputs. The resulting device is able to switch safely between autonomous and manual flight modes. The autonomous flight mode is still a hybrid mode where the cyber-pilot commands are overlapped to the manual controls. The concurrence between manual and autonomous controls also allows for the rectification of possible anomalous behaviors of the drone along the chosen trajectory, thus improving the safety. In Figure 3 we show the blocks diagram of the algorithm representing the operating logic of the mixer board.

In details, the algorithm takes as input signals the manual PPM and the autonomous contribute transmitted via UART protocol. The PPM signal is decoded in a vector of time intervals, which are expressed as integer numbers where each unit increment equals to 1 μs. Each time interval identifies a specific set-point that has been converted into the low-level flight controller format. Instead the autonomous contribute is already coded as time intervals by the Raspberry PI, in order to decrease the computational effort of the mixer and, thus, to improve the rate of the output signals. Then, we sum together the time intervals coming respectively from the manual PPM signal and the autonomous contribute, by also taking into account their sign. The resulting time intervals are finally re-encoded in a PPM signal, which is then sent to the low-level flight control board. In other terms, one can see the mixer board as an interface between the high-level navigation system (managed by the Raspberry board) and the attitude stabilization algorithm, which is implemented on the low-level controller. The main advantage of this architecture is to generate command signals directly in the standard protocol for drone flight controllers. This allows the use of any flight controller available on the market, without making any changes to the low-level firmware, thus deleting the possibility to generate catastrophic and uncontrolled errors in the code.

### 2.3. Sensors Units

The sensors units employed in this project are composed by: (i) the 6-DOF IMU connected to the Raspberry PI through the I2C serial bus, and (ii) a Raspicam camera for the purpose of computer vision. More specifically, the IMU is a Pololu AltIMU-10 v5 (Pololu Corporation, Las Vegas, NV, ~$20) that implements several standard sensors commonly used to estimate the pose of smart devices: gyroscope, accelerometer, compass, and altimeter. It is worth saying that the algorithms for position estimation, which we are going to present hereafter, only use the camera, the gyroscope and the accelerometer, but not the other sensors. Instead, regarding the Raspicam camera, it is connected to the Raspberry PI through a Mobile Industry Processor Interface (MIPI) cable.

### 2.4. Gimbal Suspension

In the following, among the novelties with respect to Reference [54], we will introduce four different algorithms to estimate the drone position. Two of them use a mechanical system to stabilize the Raspicam camera and to decouple its frame from the drone body. In particular, two different, inexpensive (price range < $120), and general purpose suspensions from the popular brand Tarot have been tested: a 2-DOF and a 3-DOF gimbal, both ensuring 0.02 degrees of precision. The former guarantees the stabilization of the pitch and roll angles, while the latter stabilizes all the three attitude angles. Each gimbal suspension is composed by: (i) two or three brushless motors to correct the attitude angles, (ii) a dedicated IMU to estimate the camera attitude, and (iii) a micro-controller to control the gimbal. These systems can behave according to different operative modes, but, roughly speaking, they act to “freeze” the camera attitude when it is close to a standing still state.

## 3. Software Modules

### 3.1. Low-Level Module: Internal Control of the System Attitude

The low-level module is made up of an open source hardware/software platform, that is, a CC3D-Revo board loaded with the LibrePilot firmware (open source software). This module has the sole task of stabilizing the attitude of the drone with respect to the set-points received from the signal mixer through the PPM protocol. Since neither the altitude nor of the position is controlled by the low-level module, another controller is required to carry out these tasks, as it will be shown in the next subsection. As a further remark, it is worth noting that the low-level flight controller allows for the hovering of the drone even in case of faults from the high-level navigation system.

### 3.2. High-Level Module: Autonomous Navigation System

The main elements of the navigation system, that is, the computer vision system, the multi-PID controller, and a Madgwick sensor fusion filter are here discussed.

Before continuing, we stress the fact that we use two different representations for the drone attitude—in Section 3.2.3, for the purpose of implementing the Madgwick sensor fusion filter, it is more convenient at the software level to describe the attitude via the quaternion formalism, while the representation with rotation matrices is adopted in Section 3.2.4 to simply get the estimate of the drone position. Since our drone is realized by means of non expensive mass market technologies, we have to prefer solutions that allow to reduce as much as possible the complexity of the software implementation.

#### 3.2.1. Computer Vision System

A computer vision algorithm is used to provide the drone with absolute references for position and attitude. This way, the drone can be accurately driven in the 3D space in a desired manner by referring the time-variation of its pose (position and orientation) to these fixed references captured from the environment.

**Figure 4:**
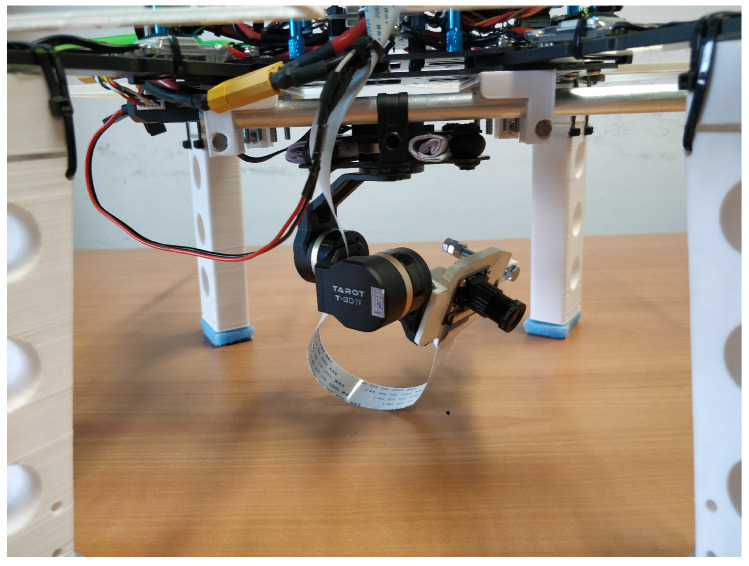
3-DOF gimbal stabilizing the Raspicam camera and thus decreasing the so-called video noise. Indeed, if we do not use the gimbal, the low refresh rate of the camera data stream is responsible for the generation of video noise contributions due to high-frequency changes of the drone attitude. Video noise implies blurred images.

In the presented UAV prototype, the computer vision system is the main element of the drone navigation system. The vision algorithm, that manages the acquisition of the images and that takes care of stitching together the acquired frames, is set to detect one or more known markers in the environment. The algorithm estimates the relative pose of the drone with respect to the markers, and, by knowing their absolute pose, is able to infer the absolute pose of the drone, as well. The camera has been tested both as a built-in device inside the body frame and mounted on a stabilized gimbal (see Section 3.2.4), as shown in Figure 4. It is worth noting that the lens frame and the one referring to the center of the thrust do not usually have the same orientation.

**Figure 5:**
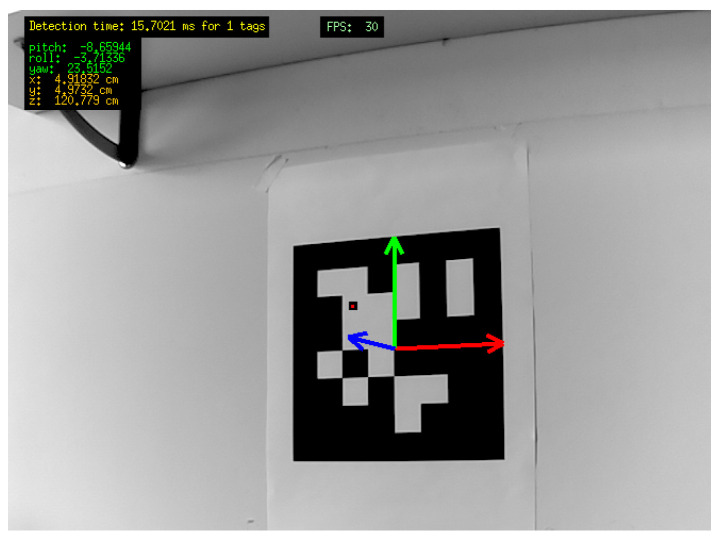
Detection and tracking process: the marker reference frame is shown in red, green and blue for *x*, *y* and *z* axes, respectively. The computer vision algorithm runs on Raspberry PI with a frequency rate of 30 Hz and provides the marker attitude and position in the lens frame [54].

The marker used in this implementation of the drone are boards featuring black and white squares. Their recognition is achieved, according to a standard practice [58], by evaluating for each pixel of the image the local magnitude of the pixels gradient, given by point-to-point differences in the pixel colour scale. Then, the gradient direction is evaluated, and pixels with similar gradient directions and magnitudes are grouped into sets by using graph-based methods. A line segment is eventually fit to each clustered pixel set. Such sets of pixels identify in the image edges from which the algorithm searches for the correct marker sequence, whose position is defined in pixel coordinates by the two-dimensional vector ${\left[u,v\right]}^{T}$, as shown in Figure 5.

The position of the marker is located in the 3D space by referring ${\left[u,v\right]}^{T}$ with respect to the lens frame. In such a frame, the coordinates

(1) $$Q_{m}\equiv {\left[x_{m},y_{m},z_{m}\right]}^{T}$$

of the marker (denoted by means of the subscript *m* in the formulas) are computed through the following relations (in this regard, see also Reference [54]) that also take into account the barrel distortion:

(2) $$\begin{array}{ccc} \frac{x_{m}}{z_{m}} & = & \frac{\left(u-u_{0}\right)\left(1+k_{ud}r^{2}\right)}{\rho_{x}} \end{array}$$

(3) $$\begin{array}{ccc} \frac{y_{m}}{z_{m}} & = & \frac{\left(v-v_{0}\right)\left(1+k_{ud}r^{2}\right)}{\rho_{y}}, \end{array}$$

where

(4) $$r^{2}=\frac{{\left(u-u_{0}\right)}^{2}}{\rho_{x}^{2}}+\frac{{\left(v-v_{0}\right)}^{2}}{\rho_{y}^{2}},$$

and ${\left[u_{0},v_{0}\right]}^{T}$ denotes the coordinates in pixel of the principal (reference) point in respect of which the image is calibrated. Instead, the parameters $\rho_{x}$ e $\rho_{y}$ are the ratio between the focal length and the size of the pixel, while $k_{ud}$ is the parameter that corrects lens distortions. Note that $k_{ud}$, $\rho_{x}$ and $\rho_{y}$ are intrinsic camera parameters, which are commonly obtained through an iterative calibration process involving the acquisition of frames of a known image in different poses. In this project, the calibration process has been performed off-line and is based on acquiring at regular time intervals at least 5 images of a chessboard with known dimension in different poses.

As further remark, observe that, if the geometrical properties (shape and dimension) of the marker are known, it is possible to retrieve additional information also with a monocular camera, such as the distance $z_{m}$ between the camera lens and the marker or the relative orientation among the marker and lens frames.

Therefore, the computer vision module is definitely able to provide information both on the position vector and on the attitude vector of the marker with respect to the drone. Hence, if the marker has a known position and attitude, also the position and orientation of the drone can be straightforwardly obtained. From here on, we will define with

(5) $$\Phi \equiv {\left[\varphi ,\theta ,\psi \right]}^{T}$$

the attitude vector of the marker with respect to the camera lens. Note that we have removed the subscript *m* from each element of Φ for the sake of simplicity of notation.

#### 3.2.2. PID-Based Control System

The autonomous driving module, that is, the core of the cyber-pilot running on the Rasperry board, computes the commands in the same form of those coming from the remote control receiver, that is, as proper reference values for roll, pitch, yaw and thrust. During the preliminary implementation stage of the project, they were conceived to maintain the drone over time in a desired position, denoted as $\bar{Q}\equiv {\left[\bar{x},\bar{y},\bar{z}\right]}^{T}$, with zero yaw relative angle $\bar{\psi }=0$, intending that the drone was facing the marker. More generally, the computer vision system provides both relative orientation and position with respect to the marker frame. This information is further integrated by means of a fusion algorithm with data coming from the on-board IMU to improve the estimate from the sole image processing, as shown in next paragraph. With the current software and hardware the computer vision system works at about 30–40 Hz, while the IMU provides data at higher frequency. The different sampling rates are managed thanks to the development of a multitasking software architecture, as explained below. The final result of the sensor fusion process is a refined estimate of the drone position $Q\equiv {\left[x,y,z\right]}^{T}$. Four different algorithms have been tested to compute *Q* with different performance.

**Figure 6:**
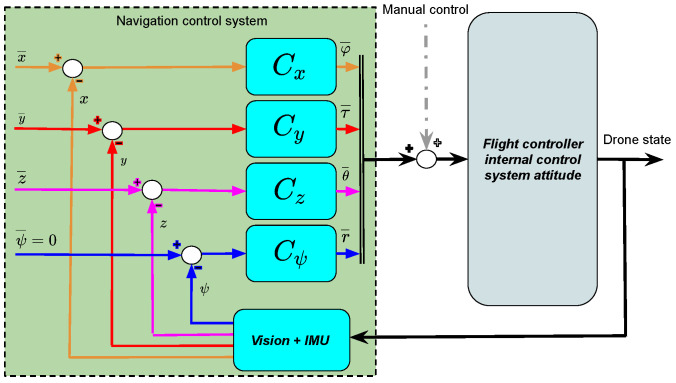
Control architecture. The navigation control system is a feedback control loop composed by four PID controllers, respectively $C_{x}$, $C_{y}$, $C_{z}$ and $C_{\psi }$ one for each pose degree of freedom, and a position estimation module fusing together stream data from the IMU and the Raspicam camera. $C_{k}$, with k∈{x,y,z}, have as input signals drone position errors and provide in output an attitude reference signal to be sent to the low-level module. Instead, $C_{\psi }$ takes in input an error signal on ψ and returns a reference for the yaw angular velocity.

As illustrated in Figure 6, the information on the errors of the drone pose, respectively given by the differences $\bar{Q}-Q$ and $\bar{\psi }-\psi$, are used to feed four distinct (decoupled) PID controllers ($C_{x}$, $C_{y}$, $C_{z}$ and $C_{\psi }$), which respectively generate the driving commands that the low-level module uses as references inputs to control roll and pitch angles, yaw angular velocity, and thrust. One can observe that the control architecture of the autonomous driver is composed by simple modules that individually act on a different pose degree of freedom. This decoupled control architecture does not directly consider the mutual interconnections among the components of the drone pose, as the fact, for example, that a change in the pitch modifies the net thrust. However, this solution for the control system has to be preferred for its reliable implementation, robustness and low computational cost. Moreover, also note that, in a first phase, each PID controller has been tuned after numerical simulations based on a simplified model of the actual drone. Then, experiments have been carried out thanks to a large number of repeated flights (about 50).

#### 3.2.3. Madgwick Sensor Fusion Filter

The navigation system implemented in the Dart drone works without explicitly modeling the dynamics of the UAV. This choice is mainly dictated by the following two reasons. First, the computational cost has not to exceed a certain threshold in order to not overload the Raspberry processor. Second, the aim is to ensure that the response of the drone to control pulses is as fast as possible, as well as the convergence of the control error.

In the chosen architecture, all the information about the pose of the drone needs to be extrapolated solely from the on-board sensors data stream. To perform the already anticipated data fusion, the Madgwick filter [59] has been chosen, since it represents the state-of-art to efficiently fuse the data coming from the accelerometer and the gyroscope within the IMU, respectively for the tracking of the translational and rotational DOFs. On one hand, the gyroscope evaluates the angular velocities of the portion of the UAV on which the IMU is mounted. The measured angular velocities are referred to the frame chosen as reference for the IMU. In principle, the corresponding orientation of the drone could be computed by integrating over time the angular velocities; however, this solution is usually highly discouraged due to the error originating from such a calculation. On the other hand, the accelerometer measures the gravitational field of the earth, taken as absolute reference. Also the information from the accelerometer is affected by noises, and this especially holds true when the sensor is moving.

The Madgwick sensor fusion filter estimates the orientation of the drone by optimally fusing the data stream from the accelerometer and the gyroscope. The orientation processed and returned by the filter is described by the quaternion representation, as for example, adopted in References [60,61]. The quaternion is a vector with four elements and generally describes the orientation of a coordinate frame with respect to another. For example, the relative orientation between the coordinate frame A and B by the angle α around the generic axis $r\equiv {\left[r_{x},r_{y},r_{z}\right]}^{T}$ can be represented by quaternion

(6) $$\begin{array}{cc} q_{B}^{A} & \equiv {\left[\cos\left(\theta /2\right),\;r_{x}\sin\left(\theta /2\right),\;r_{y}\sin\left(\theta /2\right),\;r_{z}\sin\left(\theta /2\right)\right]}^{T}\\ \; & ={\left[q_{1},\;q_{2},\;q_{3},\;q_{4}\right]}^{T}, \end{array}$$

that by definition is of unitary length. To each quaternion is uniquely associated the rotation matrix $R_{B}^{A}$ that rotates the coordinate frame A towards B according to a sequence of at least 3 rotations of the so-called Euler angles around the x,y,z axes.

In the quaternion formalism, the angular velocities measured by the gyroscope are arranged in the quaternion

(7) $$\omega_{\mathrm{IMU}}\equiv {\left[0,\omega_{x},\omega_{y},\omega_{z}\right]}^{T}\;.$$

Thus, if we solely use the data coming from the gyroscope, the orientation of the drone, expressed in the IMU coordinate frame that in turn is referred to the North-East-Down (NED) coordinates, at the *k*-th discrete time instant is equal to

(8) $${q_{g}}_{\mathrm{NED}}^{\mathrm{IMU}}\left[kT\right]=\hat{q}\left[\left(k-1\right)T\right]+{\dot{q}}_{\mathrm{NED}}^{\mathrm{IMU}}\left[kT\right]\;T\;,$$

where *T* is the sampling period, $\hat{q}\left[\left(k-1\right)T\right]$ denotes the estimate of the orientation at the previous time instant, and $\dot{q}$ is the quaternion derivative. For the specific case of the gyroscope, the quaternion derivative is just given by the quaternion product (for more details on the quaternions algebra the reader can refer for example, to References [59,60,61]) between the estimate $\hat{q}\left[\left(k-1\right)T\right]$ and the quaternion of angular velocities $\omega_{\mathrm{IMU}}\left[kT\right]$ at discrete time kT.

On the other hand, the tri-axis accelerometer evaluates the magnitude and direction of the field of gravity with respect to the sensor coordinate frame. This means that, the gravity earth field being known, the vector of measured accelerations,

(9) $$a_{\mathrm{IMU}}\equiv {\left[0,a_{x},a_{y},a_{z}\right]}^{T},$$

can be automatically referred to earth coordinate frame, here given in the NED coordinates. As conventionally taken, we assume that the direction of the gravity field is along the vertical *z* axis and is thus defined by the quaternion

(10) $$g_{\mathrm{NED}}\equiv {\left[0,0,0,1\right]}^{T}\;.$$

Then, the orientation of the accelerometer is obtained by numerically solving the following optimization problem: Minimize a cost function $f(a_{\mathrm{IMU}},g_{\mathrm{NED}},{q_{a}}_{\mathrm{NED}}^{\mathrm{IMU}})$ that identifies the distance between the vector of measured accelerations $a_{\mathrm{IMU}}$ and the direction of the gravity field $g_{\mathrm{NED}}$ rotated by the quaternion ${q_{a}}_{\mathrm{NED}}^{\mathrm{IMU}}$, the unknown parameter to be determined. The explicit analytical expression of the cost function *f* can be found in Reference [59]. Here, it is worth observing that the possibility to resort to the solution of a minimum problem comes from the evidence that the direction of the gravity field is uniquely defined in the NED coordinate frame. Thus, once measured the accelerations $a_{\mathrm{IMU}}$, one can determine the unknown quaternion ${q_{a}}_{\mathrm{NED}}^{\mathrm{IMU}}$. As proposed in Reference [59], to carry out the minimization

(11) $$\min_{q_{\mathrm{NED}}^{\mathrm{IMU}}}f\left(a_{\mathrm{IMU}},\;g_{\mathrm{NED}},\;{q_{a}}_{\mathrm{NED}}^{\mathrm{IMU}}\right)$$

in our drone we have implemented an iterative mechanism based on the gradient descent algorithm. Hence, the orientation from the accelerometer at the *k*-th discrete time instant turns out given by

(12) $${q_{a}}_{\mathrm{NED}}^{\mathrm{IMU}}\left[kT\right]=\hat{q}\left[\left(k-1\right)T\right]-\mu \left[kT\right]\frac{\nabla f}{∥\nabla f∥}\left[kT\right]\;,$$

where ∇f denotes the gradient of the geometrical surface defined by the cost function *f*, and μ is the step-size (in general, a time-dependent parameter) associated to the minimization procedure. The latter parameter determines the rate of convergence of the optimization. In this experimental work, the value of μ has been chosen constant and large in magnitude, so as to ensure that the convergence rate is equal or greater than the physical rate steering the change of the sensor orientation.

Then, the resulting estimate of the orientation provided by the Madgwick filter is obtained by fusing the orientations $q_{\mathrm{NED}}^{\mathrm{IMU}}$ (for each discrete time instant kT) as given by Equations (8) and (12), respectively from the gyroscope and the accelerometer. The fusion is practically attained according to the following relation:

(13) $$\hat{q}\left[kT\right]=\gamma \;{q_{a}}_{\mathrm{NED}}^{\mathrm{IMU}}\left[kT\right]+\left(1-\gamma \right)\;{q_{g}}_{\mathrm{NED}}^{\mathrm{IMU}}\left[kT\right]\;,$$

with γ∈[0,1]. Also the value of γ, depending on the value of μ, has been empirically chosen, so that Equation (13), which realizes the fusion of the gyroscope and accelerometer data streams, is properly balanced, that is, ${q_{a}}_{\mathrm{NED}}^{\mathrm{IMU}}$ and ${q_{g}}_{\mathrm{NED}}^{\mathrm{IMU}}$ have on average the same convergence rate. On the experimental side, this assumption leads to a quite small value of γ that privileges the stream data coming from the gyroscope with respect to the ones from the accelerometer.

**Figure 7:**
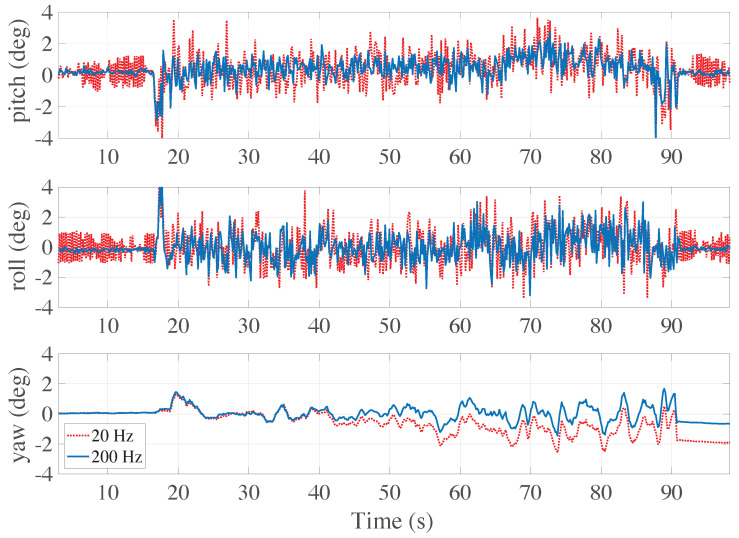
The attitude pitch, roll and yaw angles are here plotted during a flight which includes both a take-off and a hovering phase. The blue solid lines denote the output signals from the Madgwick fusion filter elaborated on board at the frequency rate of 200 Hz, whereas the red dotted lines are computed at 20 Hz.

In Figure 7 the plots of pitch, roll and yaw attitude angles, provided by the Madgwick filter implemented in Dart prototype, are reported as functions of time. In particular, the blue solid lines refer to the orientation estimates provided by the Madgwick filter where the data from the IMU is updated with a frequency rate of 200 Hz. Instead, the red dotted lines are obtained implementing the same algorithm using a frequency rate of 20 Hz. In the figure one can observe that the red and blue lines have the same phase profile, though a greater amount of noise is present in the red curves. This difference can be immediately attributed to the different values of the frequency rate sampling the IMU data stream. Similarly, it is also worth noting that a higher sampling frequency rate leads to a less pronounced drift of the yaw orientation angle, which stems from the choice of not using the magnetometer as further sensor.

#### 3.2.4. Position Estimation Methods

In this section four different methods to estimate the drone position exploiting the marker frame as reference are presented.

**Figure 8:**
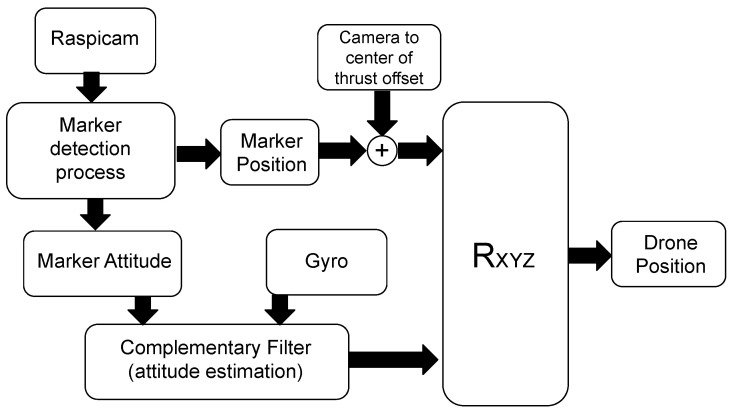
Schematic representation of the algorithm “Fixed Camera Frame Complementary Filter” (FCF-CF). The method adopts a complementary filter that fuses the stream data from the computer vision system and the gyroscope (here, the accelerator is not used). This allows to recover the information on the dynamics of the drone that is separately lost (not detected) by the two sensors. The complementary filter returns the estimate $\hat{\Phi }$ of the drone attitude vector. In this way, by rotating of such an estimated angles the position of the marker (rectified by the offset vector $Q_{\mathrm{off}}$), one can also derive the estimate of the drone position.

The first implemented algorithm—named *Fixed Camera Frame Complementary Filter* (FCF-CF) and partly presented in Reference [54]—only uses the computer vision system and the gyroscope. The schematic representation of the algorithm is depicted in Figure 8. To merge the information flows from the two sensors, we adopt a complementary filter that works according to the following relation:

(14) $${\hat{\Phi }}_{k+1}=\lambda \left({\hat{\Phi }}_{k}+T\;\Omega_{k}\right)+\left(1-\lambda \right)\Phi_{k}\;,$$

where λ is a real number belonging to [0,1], *T* is the actual sampling period, $\Phi_{k}\equiv {\left[\varphi_{k},\theta_{k},\psi_{k}\right]}^{T}$ is the attitude vector from the vision system and

(15) $$\Omega_{k}={\left[\omega_{x},\omega_{y},\omega_{z}\right]}^{T}$$

is the vector of the angular velocities around the three main drone axes coming from the gyroscope.

The new attitude estimate ${\hat{\Phi }}_{k+1}$ at the discrete time instant (k+1)T provided by Equation (14) (from now on ${\hat{\Phi }}_{k+1}$ will be abbreviated with $\hat{\Phi }$) can now be employed in the following coordinates transformation returning the estimate of the drone position:

(16) $$\begin{array}{ccc} \hat{Q} & = & \left[\begin{array}{c} x\\ y\\ z \end{array}\right]=R_{\mathrm{XYZ}}\left(\hat{\Phi }\right)\left[\begin{array}{c} x_{m}+x_{\mathrm{off}}\\ y_{m}+y_{\mathrm{off}}\\ z_{m}+z_{\mathrm{off}} \end{array}\right]\\ & = & R_{\mathrm{XYZ}}\left(\hat{\Phi }\right)\left[Q_{m}+Q_{\mathrm{off}}\right]\;. \end{array}$$

In Equation (16), the rotation matrix

(17) $$\begin{array}{ccc} & R_{\mathrm{XYZ}}\left(\hat{\Phi }\right)= & \\ & \left[\begin{array}{ccc} c_{\varphi }c_{\psi } & c_{\varphi }s_{\psi }s_{\theta }+s_{\varphi }c_{\theta } & -c_{\varphi }s_{\psi }c_{\theta }+s_{\varphi }s_{\theta }\\ -s_{\varphi }c_{\psi } & -s_{\varphi }s_{\psi }s_{\theta }+c_{\varphi }c_{\theta } & s_{\varphi }s_{\psi }c_{\theta }+c_{\varphi }s_{\theta }\\ s_{\psi } & -c_{\psi }s_{\theta } & c_{\psi }c_{\theta } \end{array}\right] \end{array}$$

brings the marker and lens frames to match, while the vector $\hat{Q}$ is the estimate of the drone position with respect to the marker frame. Instead, $Q_{\mathrm{off}}$ is a constant offset vector that takes into account the distance between the camera and the center of thrust of the drone. In this regard, by defining $Q_{\mathrm{ct}}$ as the position of the marker with respect to the center of thrust, Equation (16) can be rewritten as

(18) $$\hat{Q}=R_{\mathrm{XYZ}}\left(\hat{\Phi }\right)Q_{\mathrm{ct}}\;.$$

It is worth observing that in the FCF-CF algorithm the camera is mounted on the drone in a fixed position, which results in a persistent noise affecting the data stream coming from the camera, as a consequence of the coupling among the pose components. The resulting error is eventually amplified as the ratio between the marker distance and the camera resolution increases. However, the FCF-CF algorithm is simple to implement and has low computational cost.

**Figure 9:**
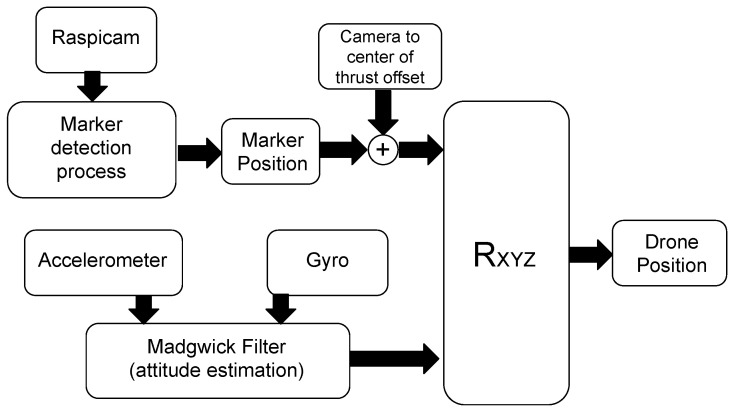
Schematic representation of the algorithm “Fixed Camera Frame Madgwick Filter” (FCF-MF). Unlike the algorithm FCF-CF, only the data stream from the accelerometer and the gyroscope are sent as input signals to the Madgwick sensor fusion filter. The latter provides an estimate of the drone orientation, whose precision does not depend on the distance between the drone and the marker.

In order to lessen the drawbacks of the first algorithm, the *Fixed Camera Frame Madgwick Filter* (FCF-MF) has been developed. In this new scenario, the Raspicam camera is still fixed with respect to the drone body, but the complementary filter has been replaced by the Madgwick filter. The schematic representation of this algorithm is shown in Figure 9. Comparing FCF-MF to the previous FCF-CF, it is worth noting that the attitude vector coming from the vision (very noisy for the coupling effects magnified by distance) is no longer necessary, since the Madgwick filter estimates it by exploiting the data from the IMU (accelerometer and gyroscope). This way, instead, the accuracy does not depend on the marker distance. Again, the attitude estimate $\hat{\Phi }$ is transformed into an estimate of the drone position through the rotation matrix $R_{\mathrm{XYZ}}$.

**Figure 10:**
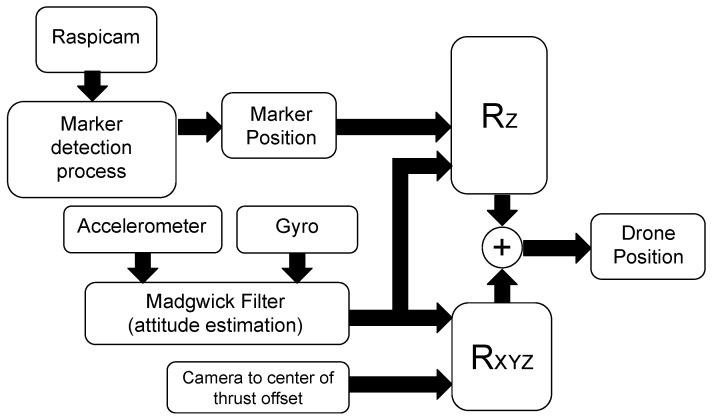
Schematic representation of the algorithm “Stabilized Camera Frame Madgwick Filter 2DOF“ (SCF-MF-2DOF). Differently to previous methods, the 2-DOF gimbal suspension is used to stabilize the video stream data with respect to the pitch and roll angles of the attitude vector. As a result, the marker coordinates have to be corrected by means of a rotation just along the *z*-axis of the estimated attitude yaw angle $\hat{\psi }$. This stabilization procedure has the advantage to reduce the noise in the video stream and thus improve the accuracy of position estimation.

The third algorithm features the Raspicam camera mounted on a 2-DOF gimbal in order to stabilize the lens frame. Thus, the position estimation method, named as *Stabilized Camera Frame Madgwick Filter 2DOF* (SCF-MF-2DOF), has been accordingly adapted. The block diagram of the SCF-MF-2DOF algorithm is shown in Figure 10.

As in the previous method, the algorithm uses the Madgwick sensor fusion filter to process the data stream from the IMU, but here $Q_{m}$ and $Q_{\mathrm{off}}$, denoting respectively the coordinates of the marker and of the center of thrust with respect to the camera (constant offset vector between the frames of the lens and the center of thrust), can rotate independently. Notice that, while $Q_{\mathrm{off}}$ must be corrected by the matrix $R_{\mathrm{XYZ}}$ defined in Equation (17) before its application to the estimate $\hat{\Phi }$, in SCF-MF-2DOF the marker coordinates need to be stabilized only around the *z*-axis of the estimated yaw angle $\hat{\psi }$ just thanks to the 2-DOF gimbal suspension. More formally,

(19) $$\hat{Q}=R_{Z}\left(\hat{\psi }\right)Q_{m}+R_{\mathrm{XYZ}}\left(\hat{\Phi }\right)Q_{\mathrm{off}},$$

where

(20) $$R_{Z}\left(\hat{\psi }\right)=\left[\begin{array}{ccc} \cos\hat{\psi } & \sin\hat{\psi } & 0\\ -\sin\hat{\psi } & \cos\hat{\psi } & 0\\ 0 & 0 & 1 \end{array}\right]\;.$$

The experiments reported in next section show that, with respect to previous methods, SCF-MF-2DOF reduces the noises and improves the accuracy almost by a factor five, thanks to the stabilization of the video stream around the pitch and roll angles by the 2-DOF gimbal.

**Figure 11:**
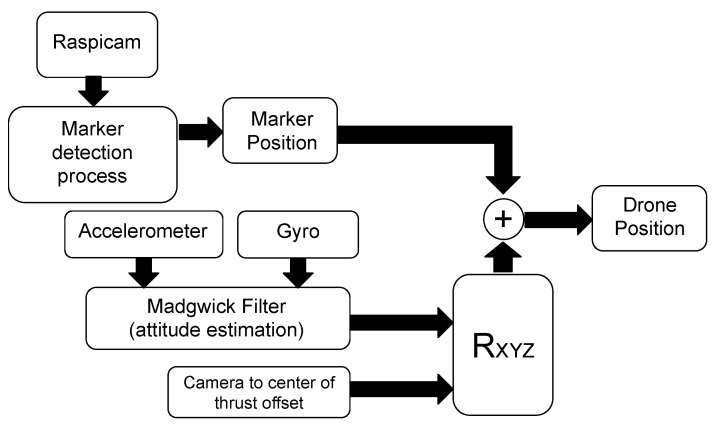
Schematic representation of the algorithm “Stabilized Camera Frame Madgwick filter 3DOF” (SCF-MF-3DOF). By improving the mechanical stabilization of the camera by means of a 3-DOF gimbal suspension, the marker coordinates $Q_{m}$ are directly summed, without being corrected, to the term $R_{\mathrm{XYZ}}\left(\hat{\Phi }\right)Q_{\mathrm{off}}$ to obtain the estimation $\hat{Q}$ of the drone position. In this way, the noise on the video stream data is further mitigated, and also less calculations are necessary to carry out the estimation procedure.

Finally, in the fourth estimation method, the 2-DOF gimbal is replaced by a 3-DOF gimbal. Hence, the marker coordinates are mechanically stabilized also with respect to the yaw angle ψ. The corresponding algorithm is here forth denoted as *Stabilized Camera Frame Madgwick filter 3DOF* (SCF-MF-3DOF). See Figure 11 for its schematic representation, whereby the block related to the correction of the marker position has been eliminated.

Accordingly, to estimate the drone position, the marker coordinates do not need to be stabilized, and, therefore, $\hat{Q}$ is just provided by the following equation:

(21) $$\hat{Q}=Q_{m}+R_{\mathrm{XYZ}}\left(\hat{\Phi }\right)Q_{\mathrm{off}}.$$

In addition to reducing the effects of noise on vision stream data, the 3-DOF gimbal has also the advantage of reducing the computational load, thus improving the precision in the estimate of both attitude and position vectors.

## 4. Tasks Architecture and Managing

**Figure 12:**
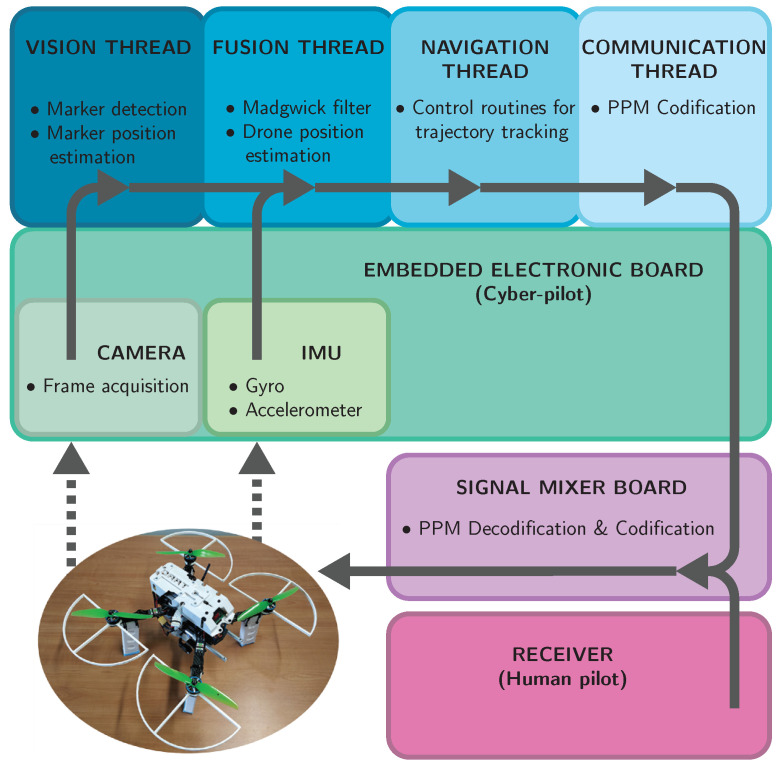
The core of the software stack is composed of four task. The vision thread is responsible for the marker detection and its position estimation from the frames acquired by the on-board camera. Its output is processed along with data from the IMU by a Madgwick filter whose aim is estimating the drone position. This latter information is then processed by the navigation thread which performs the control routines to keep the estimated trajectory as close as possible to the desired one. The navigation thread output are reference points which are transmitted to the low-level flight controller by the signal mixer, which also handles the commands of the human pilot coming from the receiver. The detailed information flow across vision and fusion threads is depicted in Figure 8, Figure 9 and Figure 10, while that of the navigation thread is shown in Figure 6.

The higher levels in the software stack of the drone navigation system comprises four distinct principal tasks. They are managed by a standard Linux scheduler set as SCHED FIFO (meaning “first input first output scheduler”), such that threads with the same priority are managed with FIFO policy. This mode can be used to implement real-time policies and it can be activated only with root permissions. It is usually adopted to reduce the time variability of the execution period of individual tasks, which is a desired feature when working with sampled processes. All the software in the higher level of the navigation system is written in C++ language and currently implemented on the Raspberry Pi 3 model B+ platform, as already described in Section 2. Figure 12 depicts how software and hardware stacks overlap.

The main task manages the computer vision system and it is responsible to carry out the processes for the marker detection and the estimation of its position and attitude. In particular, the computer vision is an aperiodic task that works at about 30 FPS (frame per second) with a resolution of 660×660 pixels. After many experimental tests on different configurations, this working condition has been found a good compromise between the number of FPS (i.e., the computational load) and the precision of the results. To improve the performance, the main task has been parallelized in four sub-tasks, one for each Raspberry core, which process part of the same frame at the same time. This way, a better use of the available calculation resources is reached, and the computations are performed faster.

The second task is a thread that manages the IMU and performs (i) the acquisition of the inertial data from the IMU itself, (ii) the Madgwick filter routine for the estimation of the drone attitude, and (iii) the drone position estimation process. Since the IMU thread is a lightweight process, it is completely lead by the IMU internal sampling time, which makes it periodic with working frequency at 200 Hz.

Instead, the third task is the thread for the control of the position trajectory: Given the desired (reference) trajectory, which in the simplest case the drone has to track point-by-point with possible constraints on the velocity profile, the control routine uses the position displacement error to generate the attitude set-points to be sent to the signal mixer. The control samples are computed only when the autonomous flight mode is activated. This control routine is managed as a periodic task forced to work at 22 Hz. The frequency is decided by the PPM protocol: Since the maximum bandwidth ensured by the PPM protocol is 44 Hz, the control routine cannot occupy more than 22 Hz, that is, half of the maximum bandwidth, so to avoid aliasing effects. However, as it will be shown in the next section on experimental results, the band of the dynamics of the drone position is comparable with the working frequency of the control task, thus making this architecture sufficient to accurately control the drone, especially when gimbal suspension are implemented. On a technical note, the integral component of the PID controllers is activated only at specific instants, that is, whenever the autonomous flight mode is enabled (event reported by a specific variable). This choice is motivated by the need to avoid discontinuities caused by the wind-up effect, when the autonomous mode is activated.

The fourth task, finally, is a communication thread which coordinates the transmission of the set-points to the mixer, that, in the autonomous mode, will send them to the flight controller. To reduce the computational load of the mixer, the attitude set-points are first converted into time intervals (see also Section 2.2) and then sent to the other boards. The communication task is a periodic routine, working at 44 Hz to exploit all the available bandwidth provided by the PPM protocol.

Eventually, a parallel task generates the mission log by saving all the data in a plain text file for offline processing. This task has the least priority and due to the access to the memory it works almost periodically at 20 Hz.

As a final technical remark, let us stress that the tasks architecture is based on a *readers-writers* synchronization method, where several tasks, which need to read shared variables (reader task), can access them simultaneously. Having implemented this distinction between readers and writers tasks, the software has better performance with respect to the standard case (*mutual exclusion* synchronization method, that is, readers and writers can access to the shared variables separately and one-at-a-time) and, thus, the data exchange process between threads is sped up.

## 5. Experimental Tests

The reference scenario used for the experimental tests presented in this section consists in tracking of a straight trajectory with triangular velocity profile. To carry out these tests, the drone takes off manually and is driven to a position where the Raspicam camera is able to identify the marker. The drone is then switched to an autonomous hovering mode (i.e., a flying mode where the reference trajectory is a point and the velocity profiles is constantly equal to zero), and finally the mission begins: The navigation software generates (on the three environmental axes *x*, *y* and *z*) a rectilinear trajectories with respect to the marker, and its tracking starts.

### 5.1. Validation of the On-Board Computer Vision System

**Figure 13:**
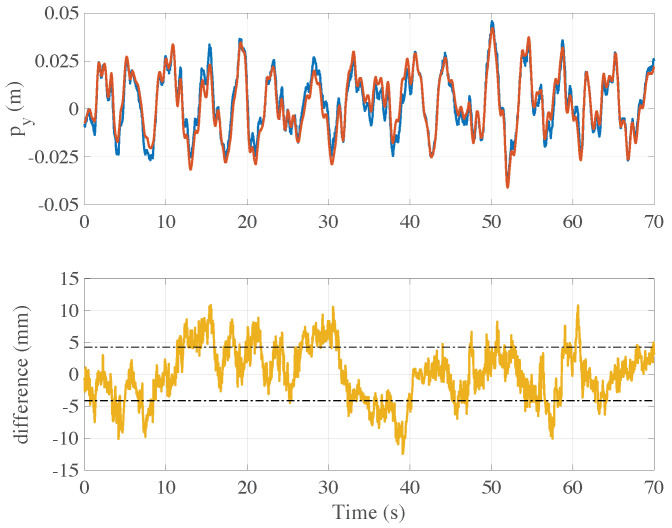
Comparison between the on-board and off-board estimations of the vision system data. In the upper panel, the estimation of the on-board altitude (blue solid line) is plotted together with the values (red solid line) measured by the fixed camera mounted on a tripod externally to the drone. Instead, the trend of the difference between the two estimates is reported in the lower panel. The mean deviation of such a difference is about 4.2 mm, while the maximum error value is around 1 cm.

Some preliminary tests were conceived to first verify the precision of the on-board computer vision system. To this aim, the drone was driven in front of a marker and set to hovering at a distance of about 4 m. The navigation algorithm computed the drone camera position (here stabilized by the 3-axis gimbal) and the related data was logged with respect to the marker inertial frame. During the experiment, a specific tag on the back of the drone camera was recorded by a high precision external camera mounted on a tripod, and the corresponding video was processed off-line by the open-source software “Kinovea”, which is able to infer the tag position with respect to the camera point of view. Such a signal, computed independently from the on-board system of the drone, was finally scaled to the marker reference system. A comparison between the off- and on-board measured trajectories along the *y*-axis (altitude) is reported in Figure 13. In the lower panel the difference between the two estimates is shown in yellow. In this experiment the standard deviation of this difference over the acquisition interval [0,70] seconds is about 4.2 mm, confirmed by other tests which witness similar values. In conclusion, the data from the on-board computer vision system turned out sufficiently informative and the very limited differences could be likely explained by the transient dynamics of the gimbal stabilization system.

### 5.2. Comparison between the Position Estimation Methods

In this subsection, the performance reached with the proposed position estimation methods and their ability to be used in a reliable virtual position sensors for the drone navigation system are discussed.

**Figure 14:**
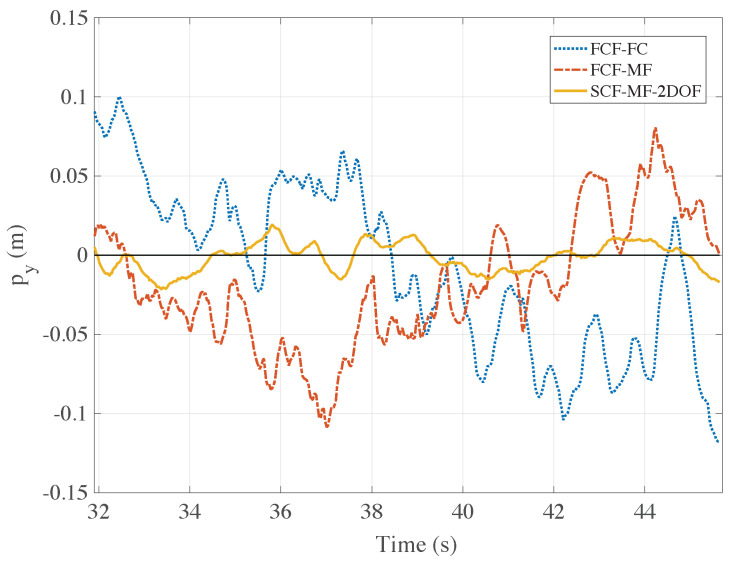
Comparison between the algorithms FCF-CF, FCF-MF and SCF-MF-2DOF for the estimation of the drone position along the altitude axis *y*. It can be observed that, if the SCF-MF-2DOF algorithm is used, the difference between the altitude estimates and the desired altitude profile ($p_{y}=0$) is of an order of magnitude smaller than the other two analyzed methods.

In a first experiment, the drone has just been set to hovering in front of a marker at 4 m distance. Given the same control system described in Section 3.2.2, the experiment has been repeated alternatively using the algorithms FCF-CF, FCF-MF, and SCF-MF-2DOF, and eventually the drone ability to hover in the right position has been investigated. In Figure 14 the drone position $p_{y}$ (altitude) as it has been estimated by the algorithms FCF-CF, FCF-MF and SCF-MF-2DOF is compared to the desired reference. As one can observe, the FCF-CF and FCF-MF algorithms achieve similar control performance, whereas the estimation yielded by the SCF-MF-2DOF method allows for an altitude profile much closer to the desired setpoint $p_{y}=0$. To clearly quantify the performance of the algorithms, in Table 1 we provide the *corrected sample standard deviation $s_{\varepsilon }$* of the error ε computed as the difference between the estimated drone position and the desired trajectory within the time interval under investigation. The sample standard deviation is defined as

(22) $$s_{\varepsilon }\equiv \sqrt{\frac{1}{N-1}\sum_{k=1}^{N}{\left(\varepsilon -\bar{\varepsilon }\right)}^{2}},$$

where N=50 is the number of performed experimental tests and $\bar{\varepsilon }\equiv \sum_{k=1}^{N}\varepsilon /N$.

As shown in the table, the performance of the algorithm SCF-MF-2DOF are much better than the ones of the first two proposed estimation methods and, quantitatively, the sample standard deviations $s_{\varepsilon }$ of the error are halved.

**Figure 15:**
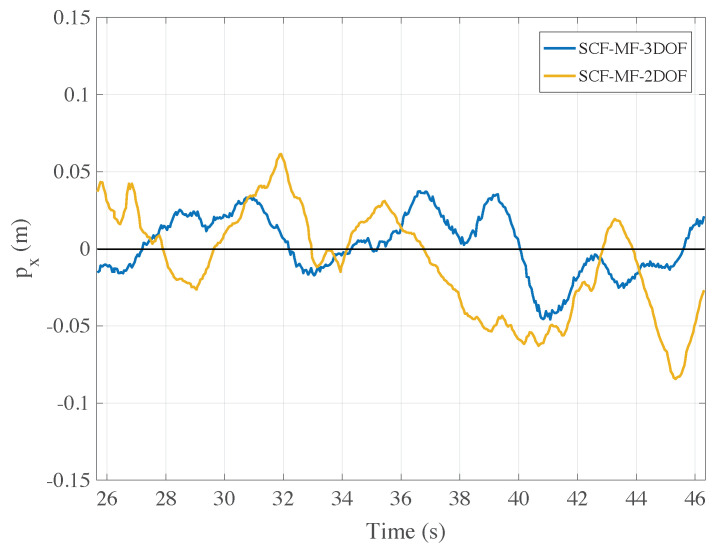
Comparison between the algorithms SCF-MF-2DOF (with 2-DOF gimbal) and SCF-MF-3DOF that uses a 3-DOF gimbal suspension for the stabilization of the yaw angle. In this case, the comparison is made between the estimates obtained along the horizontal axis *x*.

In Figure 15 it is reported, for a similar experiment, the comparison between the estimation algorithms SCF-MF-2DOF and SCF-MF-3DOF taking this time into account the horizontal position $p_{x}$.

### 5.3. Autonomous Flight Test

Indeed, in this latter case the camera yaw angle is now stabilized by the use of the 3-DOF gimbal suspension and is always pointing at the same direction. In terms of tracking precision, the performance of the SCF-MF-3DOF algorithm has to be mainly evaluated along the horizontal axis *x*, being unchanged for the other axes. The sample standard deviation $s_{\varepsilon }$ of the estimation errors for the algorithms SCF-MF-2DOF and SCF-MF-3DOF have been computed again by repeating N=50 times the same experiments with fixed working conditions. The values of the error standard deviations are provided in Table 2. 

From the table, the error along the horizontal axis turns out larger than the one along the vertical axis. Nevertheless, the standard deviation of the error is about 2.5 cm, thus proving overall a few centimeter precision in controlling the position of the Dart drone prototype when exploiting the SCF-MF-3DOF on-board navigation system.

**Figure 16:**
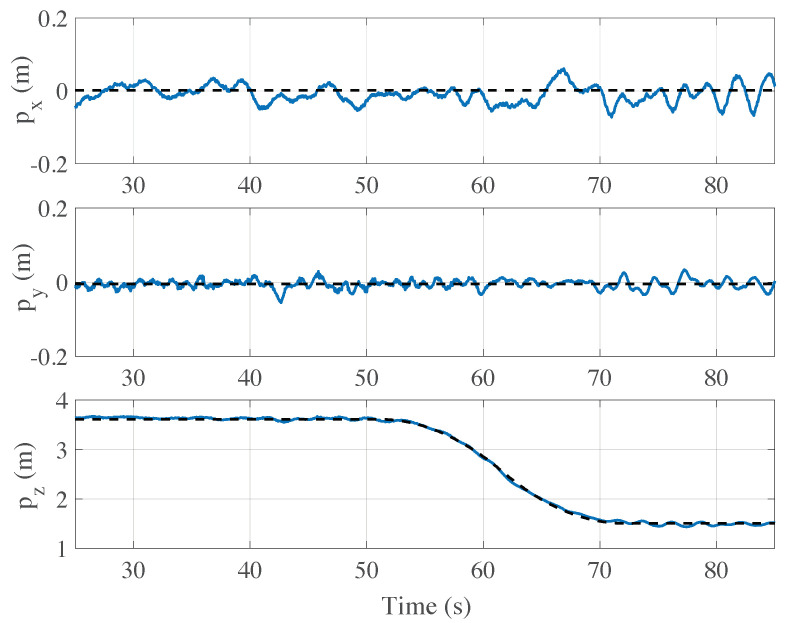
Drone position vs time: the blue solid curves represent the 3D-position of the drone in an experimental test with a desired straight trajectory having a triangular velocity profile (black dashed lines) starting at t=50 s. The estimated positions of the drone are obtained on-board applying the implemented SCF-MF-3DOF algorithm to the data from the IMU and the camera during the flight.

In Figure 16, we show the time-behaviour of the drone position (blue solid lines for the elements $p_{x},p_{y},p_{z}$) while the drone is in the autonomous flight mode, during the tracking of a preset rectilinear trajectory. The measured position of the drone is also compared with the desired trajectory (black dashed lines) that has to be tracked. During the time evolution in the interval [25,50] seconds, the autonomous hovering mode is enabled, which results in having an almost constant value of the 3D-position. Instead, from t=50 s until the end of the test, the autonomous flight mode was enabled, so as to allow for the tracking of a straight trajectory with a triangular velocity profile. In the test, the maximum value of the velocity is 0.1 m/s. This implies a variation of the desired trajectory (blue curve) along *z*. The mission ends when the drone reaches a distance of 1.5 m away from the marker. At that distance, the drone automatically returns to the autonomous hovering mode and stops moving.

Both the hovering and autonomous modes mainly use the data stream from the computer vision system and from the IMU. Although signals from the sensors are filtered from external noise sources and fused together, we are able to achieve a correct tracking of the trajectory along the three axes but with a self-sustained oscillation perceptible on $p_{x}$ and $p_{y}$. Such oscillations are originated by a delay of around 0.2 s in the video acquisition process (due to data buffer) and affects the performance of the navigation control system. This aspect, that has been already partially discussed in Reference [46], will be properly addressed in future research.

## 6. Conclusions

In this paper, a prototype of UAV, able to autonomously track 3D trajectories, has been presented. In order to have easy and complete access to all the parts of the system, the drone has been developed from scratch by using only “standard” components, that is, inexpensive equipment already present in the mass market. The developed solutions, however, can be applied to off-the-shelf drones as well, at least to the class of professional UAVs that can support a reasonable payload. Indeed, the core idea of the proposed technology for autonomy is the “cyber-pilot”, that is, a vision based navigation system, exploiting only on-board devices, which can substitute the human commands in driving the drone along a desired trajectory with high precision. The additional hardware necessary for the cyber-pilot is made of two programmable embedded boards (Raspberry Pi 3B+ and Arduino Nano), a small camera (Raspicam), a 6-DOF IMU, and, possibly, a gimbal (2DOF or 3DOF). A rough estimate of the cost ranges from $80 to $200 depending of the actual market prices and on the chosen configuration (with or without gimbal). This hardware is used to implement a “virtual sensor”, that is, a sensor fusion algorithm which merges data from the IMU and the on-board computer vision system. The resulting information is exploited by a simple control logic which makes the navigation system override human commands when it is in autonomous mode. Experiments suggest that low-cost technologies, such as the ones used to implement the UAV, are very close to enable the sought passage from meter- to centimeter-scale precision in autonomous maneuvering of multi-rotor drones that would represent a noteworthy generation change in their application range. Moreover, both the hardware and the software proposed architectures are modular and they can easily be extended and enhanced, for instance, by replacing more refined algorithms into the programs, or by substituting a device with a better performing equipment. Such a feature is crucial for the maintenance of the project. Indeed, new modules are actually under working to update the drone thanks to novel devices, which have recently win over the mass market.

## Figures and Tables

**Table 1:** Standard deviation of the altitude position error for different algorithms.

Algorithm	Standard Deviation (Along *y*)
FCF-CF	5.35 cm
FCF-MF	4.01 cm
SCF-2DOF	1.77 cm

**Table 2:** Standard deviation of the position error along the *x*-axis for different algorithms.

Algorithm	Standard Deviation (Along *x*)
SCF-2DOF	3.42 cm
SCF-3DOF	2.54 cm

## Data Availability

All experimental data will be made available on request to the corresponding author with appropriate justification.

## Abbreviations

The following abbreviations are used in this manuscript:

DOF	Degrees of freedom
IMU	Inertial measurement unit
UAV	Unmanned aerial vehicle
PID	Proportional–integral–derivative
PPM	Pulse position modulation
NED	North-east-down
FIFO	First input first output
FPS	Frame per second
FCF-CF	Fixed camera frame complementary filter
FCF-MF	Fixed camera frame Madgwick filter
SCF-MF-2DOF	Stabilized camera frame Madgwick filter 2DOF
SCF-MF-3DOF	Stabilized camera frame Madgwick filter 3DOF
